# Intrapopulation changes in Puccinia hordei induced by two-component fungicides from different chemical classes

**DOI:** 10.18699/vjgb-25-126

**Published:** 2025-12

**Authors:** M.S. Gvozdeva, O.A. Kudinova, V.D. Rudenko, G.V. Volkova

**Affiliations:** Federal Research Center of Biological Plant Protection, Krasnodar, Russia; Federal Research Center of Biological Plant Protection, Krasnodar, Russia; Federal Research Center of Biological Plant Protection, Krasnodar, Russia; Federal Research Center of Biological Plant Protection, Krasnodar, Russia

**Keywords:** barley, leaf rust of barley, fungicides, Puccinia hordei, resistance, pathogenicity, population, ячмень, карликовая ржавчина, фунгициды, Puccinia hordei, резистентность, патогенность, популяция

## Abstract

Fungicide resistance is a global problem that reduces the effectiveness and duration of action of these compounds due to changes in the racial composition and virulence of phytopathogen populations. Currently, resistance to 100 active substances has been registered in more than 230 fungal plant pathogens. Leaf rust of barley (Puccinia hordei Otth.) is one of the most widespread and harmful pathogens in the barley pathocomplex; it is recorded in southern Russia every year. There are very few studies on the effect of fungicides on the characteristics of rust fungi populations, and none have been carried out on P. hordei in Russia. This research aimed to analyze the effect of fungicides belonging to the chemical classes of triazoles and strobilurins on intrapopulation changes in P. hordei in terms of pathogenicity (virulence and aggressiveness) under the conditions of the North Caucasus region of Russia. Two-component fungicides approved for use in the Russian Federation were selected for the study: Delaro, SC; Amistar Extra, SC; Amistar Gold, SC. Plants were treated using several application rates: 50, 100, 150 and 200 % (the recommended application rate was determined to be 100 %). Treatment of winter barley plants with fungicides with different application rates revealed intrapopulation changes in the virulence structure of P. hordei. In all treatment variants, the frequency of isolates virulent to the Rph4, Rph5, Rph6+2, Rph12 genes decreased with increasing fungicide application rate and the frequency of isolates virulent to Rph14 increased. No isolates virulent to Rph7 were found in either the original population or the experimental variants. The average virulence of the fungal populations treated with the fungicides in all experimental variants was lower compared to the original population (no treatment (48.5 %)) and depending on the application rate varied from 33.8 % (Amistar Gold, 50 %) to 28.5 % (Amistar Gold, 200 %). Under the influence of the increased application rates of the fungicides, an increase in the duration of the latent period was observed: from 168 h (original population) to 216 h (Delaro, Amistar Gold, 200 %). A decrease in sporulation ability (spore mass per pustule ranged from 0.013 mg (original population) to 0.002 mg (Delaro, Amistar Gold, 200 %)) and in the viability of P. hordei (from 100 % for the original population to 22.5 % in Amistar Gold, 200 % treatment) was found under the action of the fungicides. Thus, a fungicide-treated P. hordei population is characterized by intrapopulation changes in aggressiveness and virulence, which can significantly increase barley yield losses due to a decrease in the effectiveness of chemical protection, as well as an increase in the harmfulness of the pathogen.

## Abstract (ru)

Резистентность к фунгицидам является мировой проблемой, обуславливающей снижение их эффективности и срока действия вследствие изменения расового состава и вирулентности популяций фитопатогенов. В настоящее время зарегистрирована резистентность более чем у 230 грибных патогенов растений к 100 действующим веществам. Возбудитель карликовой ржавчины (Puccinia hordei Otth.) – один из распространенных и вредоносных в патокомплексе ячменя, в Южном федеральном округе, фиксируется на посевах ежегодно. Исследований по влиянию фунгицидов на характеристики популяции ржавчинных грибов крайне мало, а в отношении P. hordei в России не проводилось. Цель данной работы – проанализировать влияние фунгицидов из химических классов триазолов и стробилуринов на внутрипопуляционные изменения P. hordei по показателям патогенности (вирулентности и агрессивности) в условиях Северо-Кавказского региона России. Для исследования были выбраны двухкомпонентные фунгициды, разрешенные к применению на территории Российской Федерации: Деларо, КС; Амистар Экстра, СК; Амистар Голд, СК. Обработку растений проводили с использованием нескольких норм применения препаратов: 50, 100, 150 и 200 % (рекомендуемая норма применения принята за 100 %). При обработке растений ячменя озимого фунгицидами с различной нормой применения выявлены внутрипопуляционные изменения в структуре P. hordei по вирулентности. Во всех вариантах с увеличением нормы применения фунгицидов происходили снижение частоты изолятов, вирулентных к генам Rph4, Rph5, Rph6+2, Rph12, и увеличение частоты изолятов, вирулентных к Rph14. Не обнаружено изолятов, вирулентных к Rph7, как в исходной популяции, так и в вариантах опыта. Средняя вирулентность популяций гриба, обработанных фунгицидами, во всех вариантах опыта была ниже по сравнению с вирулентностью исходной популяции (без обработки) (48.5 %) и в зависимости от нормы применения варьировала от 33.8 (Амистар Голд, 50 %) до 28. 5 % (Амистар Голд, 200 %). Под действием повышенных норм применения препаратов отмечено увеличение длительности латентного периода развития инфекции – от 168 ч (исходная популяция) до 216 ч (Деларо, Амистар Голд, 200 %). Под действием фунгицидов установлено снижение спорулирующей способности (масса спор с одной пустулы варьировала от 0.013 (исходная популяция) до 0.002 мг (Деларо, Амистар Голд, 200 %) и жизнеспособности P. hordei (от 100 % для исходной популяции до 22.5 % при обработке Амистар Голд, 200 %). Таким образом, популяция P. hordei, подвергавшаяся обработке фунгицидами, характеризуется внутрипопуляционными изменениями по показателям агрессивности и вирулентности, что может существенно увеличивать потери урожая ячменя вследствие как снижения эффективности химической защиты, так и повышения вредоносности патогена.

## Introduction

Southern Russia is the leader in terms of winter grain crops area. According to the Federal State Statistics Service (https:// rosstat.gov.ru/storage/mediabank/posev-4сх_2024.xlsx), in 2024 their share in total crops was 51.3 %. Winter barley is a promising crop, valuable for livestock farming, capable of producing a consistently high yield even in the extremely dry conditions of southern Russia (Ereshko et al., 2012). In 2024, the Krasnodar Region was the leader in barley harvesting: 1,159.5 thousand tons were harvested, which is 7 % of the total amount in the country (https://graininfo.ru/news/yachmenploshchadi- sbory-i-urozhaynost-v-rossii-v-2024-godu/).

Barley crops are affected by various pathogens that cause yield failure and loss of grain quality (Abbas, 2022). The leaf rust of barley pathogen, the biotrophic basidiomycete Puccina hordei Otth., is one of the widespread and harmful pathogens in the barley pathocomplex (Sapkota et al., 2023). In the North Caucasus region of Russia, leaf rust on barley crops is recorded annually, and epiphytoties occur once or twice every 10 years (Volkova et al., 2018). Ensuring high yields is impossible without chemical protection. In addition to the high pesticide load on agrobiocenosis, a serious problem is the emergence of pathogen forms resistant to the active substances of fungicides (Shcherbakova, 2019).

Fungicide resistance has become a major global problem, reducing the efficacy and shelf-life of some promising fungicides (Brent, Hollomon, 1995; Thind, 2021). Currently, resistance to 100 active substances has been reported in more than 230 fungal plant pathogens in various crops and geographic regions (FRAC, 2020). According to P.E. Russell (2003), rust fungi are pathogens with a low risk of emerging resistance, but their rapid life cycle, airborne dispersal of spores, and mixed mode of reproduction can cause intrapopulation changes (Ji et al., 2023) due to the rapid spread and accumulation of resistant forms, which will lead to decreased sensitivity to fungicides.

Traditionally, fungicides of the triazole and strobilurin classes are used to control rust diseases of wheat and barley (Walters et al., 2012). Triazoles belong to the largest class of fungicides with the demethylation inhibitor (DMI) group, suppressing the biosynthesis of ergosterol, a key component of the plasma membrane of fungal cells (Lass-Flörl, 2011). According to FRAC (2020), there is a moderate risk of emerging resistance to such fungicides. At the same time, a number of studies have noted changes in the population structure of biotrophic pathogens. For example, G. Zhan et al. (2022), studying the susceptibility to triademiphon in 446 isolates of Puccinia graminis, found that fungicide-resistant isolates showed strong adaptive traits in terms of urediniospore germination rate, latent period, sporulation intensity and lesion spread rate.

Strobilurins are an equally large class of fungicides that accumulate in the waxy layer of the leaf cuticle after plant treatment (Krupen’ko, 2023). Globally, strobilurins account for 20–25 % of total fungicide sales, a third of which is attributed to azoxystrobin, the best-selling fungicide in the world (Leadbeater, 2012). The first strobilurin-resistant isolates were detected in 1998 in Blumeria graminis [DC.] in Germany two years after their use, and strobilurin resistance has now been reported among both biotrophic (Dodhia et al., 2021) and hemibiotrophic (Ölmez et al., 2023) pathogens worldwide.

There is also a risk of emerging fungicide resistance for barley leaf rust (Walters et al., 2012). Like all rust fungi, P. hordei is a rapidly evolving phytopathogen (Çelik Oğuz, Karakaya, 2021). There are very few studies on the effect of fungicides on the characteristics of the rust fungi population. For the first time in Russia, such work was carried out at the Federal State Budgetary Scientific Institution “Federal Research Center of Biological Plant Protection” (FSBSI FRCBPP) for the “wheat–yellow rust pathogen” pathosystem (Volkova, 2007). The effect of a triadimefon-based fungicide on the virulence and aggressiveness of P. striiformis was studied, the rate of emergence of resistant forms of the pathogen was calculated, and an anti-resistant strategy for the use of fungicides with this active substance on wheat crops was developed and proposed. Further studies were carried out on the wheat leaf rust pathogen (Gvozdeva, Volkova, 2022). It was found that the population of P. triticina treated with a chemical fungicide based on tebuconazole is characterized by a change in the structure of aggressiveness and virulence and a decrease in sensitivity to the toxicant. Similar studies on barley leaf rust have not been carried out either in the world or in Russia. Given the high virulence and variability of the North Caucasian population of the pathogen (Danilova, Volkova, 2022), and the need to develop an anti-resistant strategy for each pathogen and cenosis (Corkley et al., 2021), research on this issue is extremely relevant

This research is aimed at analyzing the effect of fungicides of the chemical classes of triazoles and strobilurins on intrapopulation changes in P. hordei in terms of pathogenicity (virulence and aggressiveness) under the conditions of the North Caucasus region of Russia

## Materials and methods

The studies were carried out in the laboratory and greenhouse of the FSBSI FRCBPP on the winter barley variety Vivat, susceptible to leaf rust. Originator of the variety is FSBSI “Donskoy Agrarian Scientific Center”; it is recommended for growing in the North Caucasus region and resistant to lodging and frost.

To obtain spore material of the North Caucasian population of the barley leaf rust pathogen (further in the text – fungal urediniospores, or population), infected leaves were collected during a route survey of industrial barley crops in the Krasnodar Region, Stavropol Region, Rostov Region and the Republic of Adygea. Then, the susceptible Vivat variety was inoculated with a mixture of herbarium samples (Fungal Pathogens of Grain Ear Crops…, 2024). The spore material collected in the required quantity was stored at a temperature of +4 °C.

Two-component fungicides approved for use in the Rus- sian Federation were selected for the study: Delaro, SC (175 g/l prothioconazole + 150 g/l trifloxystrobin); Amistar Extra, SC (200 g/l azoxystrobin + 80 g/l cyproconazole); Amistar Gold, SC (125 g/l azoxystrobin + 125 g/l difenoconazole).

Inoculation of winter barley plants with a spore suspension of the pathogen was carried out in the seedling phase. The infected plants were kept in a humid chamber for 18 hours, then they were grown under controlled conditions at a temperature of +20–22 °C, air humidity of 70–80 %, and light intensity of 10–15 thousand lux with a day and night cycle (16/8 hours) (Fungal Pathogens of Grain Ear Crops…, 2024). When the first signs of the disease appeared, spraying was carried out using several application rates of the preparations: 50, 100, 150 and 200 % (the recommended application rate was determined to be 100 %).

Urediniospores of P. hordei, collected from barley plants treated with fungicides at various application rates, were transferred to intact plants for reproduction and determination of aggressiveness indices (spore viability, latent period duration, sporulating capacity, sporulation duration). Aggressiveness indices were determined for the mixed pathogen populations obtained as described above

The viability of barley leaf rust spores was tested in a humid chamber under a microscope by counting the total number of fungal spores and germinated spores (Sanin et al., 1975). The duration of the latent period was counted from the moment of inoculation until the first signs of the disease appeared (Pyzhikova, 1972). The sporulating capacity was determined by calculating the ratio of the number of pustules to the mass of the collected biomaterial. The duration of sporulation was determined from the beginning of the pustules opening until the end of sporulation (Sanin et al., 1975).

The virulence of the P. hordei populations treated with fungicides at different application rates was determined by the reaction of 15 barley differentiators from the International and Australian sets containing known genes of resistance to the pathogen: Sudan (Rph1), Peruvian (Rph2), Estate (Rph3), Gold (Rph4), Magnif 104 (Rph5), Bolivia (Rph6+2), Cebada Capa (Rph7), Egypt 4 (Rph8), Abyssinian (Rph9), Triumph (Rph12), PI 531849 (Rph13), PI 584760 (Rph14), Prior (Rph19), Ricardo (Rph21+2), Fong Tien (Rph25). The infection types were recorded 10–12 days after inoculation using the Levin and Cherevik scale in points (Volkova et al., 2018). The frequencies of virulence genes were calculated using the probabilistic method (Wolfe, Schwarzbach, 1975) based on the ratio of the number of pustules with infection type of 3–4 points on the lines with known resistance genes to the number of pustules on the susceptible variety Vivat. To evenly distribute P. hordei spores on barley leaves, inoculation was carried out in a precipitation tower using lycopodium, which was mixed with fungal spores in a ratio of 400:1 (Parlevliet, 1980).

Based on the results of differentiation, the average virulence was calculated (Mihajlova et al., 2003). Differences between the original population (without treatment) and populations treated with different application rates of the preparation in terms of virulence gene frequencies were determined using the Nei index (Nei, 1972). The statistical reliability of the aggressiveness indicators was assessed using the Fisher criterion (α = 0.05) using STATISTICA 10.0 software.

The research used the material and technical base of the Unique Scientific Installation (USI) “Phytotron for Isolation, Identification, Study and Maintenance of Races, Strains, Phenotypes of Pathogens” (https://fncbzr.ru/brk-i-unu/uniqueinstallation- 2/) and objects of the bioresource collection of the Federal State Budgetary Scientific Institution FNCBZR “State Collection of Entomoacariphages and Microorganisms” (https://fncbzr.ru/brk-i-unu/unique-installation-1/).

## Results

When treating winter barley plants with fungicides at different application rates, intrapopulation changes in the structure of P. hordei by virulence were revealed (Table 1)

**Table 1.**
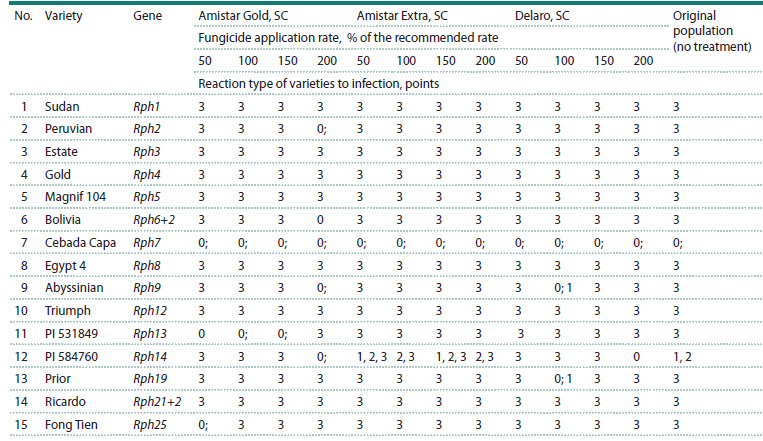
Infectious type of differentiating varieties in response to inoculation with P. hordei population under the influence of various fungicide application rates (greenhouse of the FRCBPP, 2024)

In the option with the Amistar Gold, SC preparation, with an increase in the rate to 200 % of the recommended one, a decrease in the virulence of the pathogen was observed on varieties containing the resistance genes Rph2, Rph6+2, Rph9, Rph14. On the line with the Rph13 and Rph25 genes, when treating the barley plants with the preparations at an application rate of 50 % of the recommended one, no damage was observed. With an increase in the rate to 200 %, the type of reaction to infection increased to 3 points

Treatment of the barley plants with the fungicide Amistar Extra, SC affected the fungus population towards increasing virulence to the Rph14 gene, while a heterogeneous infection type was observed, pustules of different sizes, areas of dead and chlorotic tissue were present on the leaf.

In the option with the fungicide Delaro, SC, a decrease in the virulence of the P. hordei population to the Rph14 gene was noted. The reaction type decreased from 3 (application rate 50 % of the recommended one) to 0 points (application rate 50 % of the recommended one), for the original population this indicator was 1 and 2 points. When using the recommended application rate of the preparation (100 %), the infection type of the varieties containing the resistance genes Rph19 and Rph9 was 0;, 1 point; for the original population this indicator was 3 points.The type of damage to the varieties containing the resistance genes Rph1, Rph3, Rph4, Rph5, Rph7, Rph8, Rph12, Rph21+2, in all options with the fungicides, regardless of the application rate, corresponded to the type of the original population (no treatment) and amounted up to 3 points.

This research studied the effect of the fungicides on the virulence of the population of the barley leaf rust pathogen (Table 2).

**Table 2.**
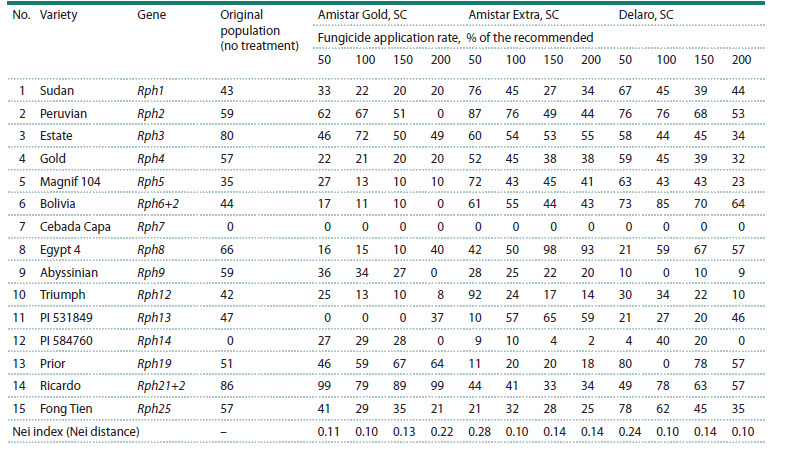
Virulence (%) of the P. hordei population treated with the fungicides at different application rates to lines carrying Rph genes (greenhouse of the FSBSI FRCBPP, 2024)

In all experimental options with an increase in the rate of fungicide application, there was a decrease in the virulence of the fungal population to Rph4, Rph5, Rph6+2, Rph12; in the option with Amistar Extra, SC and Delaro, SC – to Rph2; Amistar Gold, SC and Amistar Extra, SC – to Rph9. The use of the studied fungicides at a rate of 50–150 % of the recommended one contributed to an increase in the virulence of the population to Rph14. Under the influence of fungicides Amistar Gold, SC and Delaro, SC with an application rate of 150 % of the recommended one, an increase in the virulence of the population to Rph19 was noted. Treatment of plants with Amistar Extra, SC and Delaro, SC with an increase in the application rate to 150 % of the recommended one contributed to a decrease in the virulence of the population to Rph1, and an increase in the occurrence of isolates virulent to Rph8.

According to the Nei index, the maximum differences in the frequency of isolates virulent to lines with Rph genes were obtained between the original population (no treatment) and the population treated with Amistar Extra, SC and Delaro, SC with an application rate of 50 % of the recommended one (N = 0.28; N = 0.24, respectively). At the same time, in the option with the fungicide Amistar Gold, SC, the maximum differences were obtained when using an application rate of 200 % of the recommended one (N = 0.22).

**Fig. 1.**
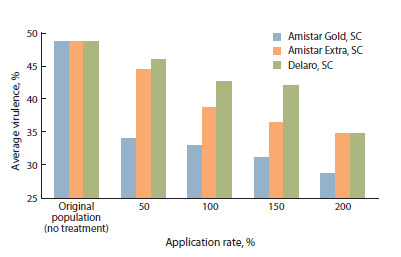
Average virulence of the population of the barley leaf rust pathogen under the influence of different rates of fungicide application (%) (laboratory and greenhouse of the FSBSI FRCBPP, 2024).

The average virulence of the original P. hordei population (no treatment) was 48.4 % (Fig. 1). In all experimental options, with an increase in the rate of fungicide application, a change in the average virulence was noted.

Treatment of the plants with Delaro, SC contributed to a decrease in this indicator from 45.9 % (application rate 50 % of the recommended one) to 34.7 % (application rate 200 %), with Amistar Extra, SC – from 44.3 % (application rate 50 % of the recommended one) to 34.7 % (application rate 200 %), with Amistar Gold, SC – from 33.8 % (application rate 50 % of the recommended one) to 28.5 % (application rate 200 %).

The results of the influence of different fungicide application rates on the aggressiveness indices of barley leaf rust were obtained. An increase in the duration of the latent period of the disease under the influence of high application rates of preparations was noted. For the original population (no treatment), this indicator was 168 hours, which corresponded to the values obtained when treating barley plants with the studied fungicides at a reduced application rate (50 % of the recommended one). In the option with the fungicides Delaro, SC and Amistar Gold, SC, at an application rate of 200 % of the recommended one, the duration of the latent period increased to 216 hours, with Amistar Extra, SC, up to 192 hours.

A decrease in the sporulating capacity of the P. hordei population treated with fungicides was found. In the options with the preparations Delaro, SC and Amistar Gold, SC, with an increase in the application rate, the spore mass from one pustule decreased from 0.007 (application rate 50 % of the recommended one) to 0.002 mg (application rate 200 %). In the option with the fungicide Amistar Extra, SC, this indicator changed from 0.006 (application rate 50 % of the recommended one) to 0.005 mg (application rate 200 %). For the original population (no treatment), the sporulating capacity was 0.013 mg from one pustule.

**Fig. 2.**
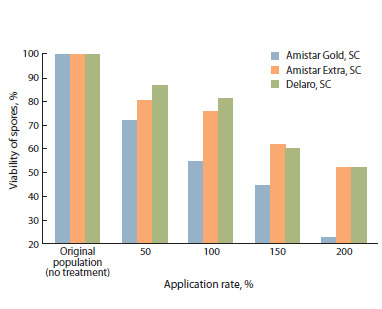
Influence of different application rates of the fungicides on the viability of barley leaf rust spores (laboratory and greenhouse of the FSBSI FRCBPP, 2024).

A change in the viability of P. hordei spores was noted to be influenced by the fungicides. Thus, for the original population (no treatment), the value of this indicator was determined as 100 % (Fig. 2). In the option with the fungicide Delaro, SC, the viability of spores decreased from 86.7 (application rate 50 % of the recommended one) to 51.7 % (application rate 200 %); in the option with the preparation Amistar Extra, SC, from 80.0 (application rate 50 % of the recommended one) to 51.7 % (application rate 200 %); with Amistar Gold, SC, from 71.7 (application rate 50 % of the recommended one) to 22.5(application rate 200 %). 

## Discussion

The results obtained during the research are consistent with the studies of other scientists. Thus, C. Zhao et al. managed to obtain two flutolanil-resistant isolates of Rhizoctonia spp., which were characterized by a lower mycelial growth rate and reduced virulence towards sugar beet sprouts compared to the original isolate (Zhao et al., 2019).

An inhibitory effect on the sporulating ability of the Phytophthora infestans fungus was noted with an increase in the application rate of the fungicides with difenoconazole and fludioxonil as active ingredients (Myca, 2015).

Under the influence of an aqueous solution of benzimidazole (40 mg/l), a decrease in the sporulating capacity and the number of pustules of the rye leaf rust pathogen P. dispers was determined in comparison with the control, and the avirulence of the fungus was also noted (Tyryshkin, 2017).

G.V. Volkova’s studies (2007) have proven that the acquisition of resistance to a fungicide from the triazole class was accompanied by a decrease in sporulating capacity by 4.8 times, the rate of diffuse spread of the pathogen mycelium in the tissues of the host plant, by 4 times. The incubation period was extended by 3 days, the period of pustule formation, by 8–12 days.

Previously, data were obtained on the effect of a tebuconazole- based fungicide on the aggressiveness of the population of the wheat leaf rust pathogen. With an increase in the application rate of the preparation, the viability of spores decreased to 21.5 %, the sporulating capacity, to 0.02 mg of spores, and the duration of sporulation, to 8 days (application rate 0.7 l/ha). At the same time, the duration of the latent period increased to 233 hours (Gvozdeva, Volkova, 2022).Changes in the population structure under the influence of triazole fungicides have been recorded. Thus, X. Wu et al. (2020) described the sensitivity of 89 P. graminis isolates to triademifon, a triazole fungicide. It was found that isolates with resistance to triademifon may have cross-resistance to carbendazim. Resistant isolates to azole fungicides have also been recorded in P. striiformis (Tian et al., 2019), B. graminis (Cao et al., 2008). For the P. triticina population from Brazil, a decrease in sensitivity to triazoles was noted for the five most common races of the pathogen (Ardium et al., 2012). We studied the change in the P. triticina population structure under the influence of a tebuconazole-based fungicide (Gvozdeva, Volkova, 2022). According to the data obtained, the maximum of the studied changes in the genetic structure of the population (according to Nei, N) were noted at reduced rates of fungicide application. The average virulence of the pathogen population decreased with an increase in the rate of fungicide application

Under the influence of the studied fungicides, a decrease in the virulence of the fungal population to Rph4, Rph5, Rph6+2, Rph12 was found, which have been ineffective for the North Caucasian population for more than 10 years (Volkova et al., 2019; Danilova, Volkova, 2023). At the same time, virulence to Rph14 increased compared to the original population. In 2021, the frequency of fungal isolates to this line was low (Danilova, Volkova, 2023). This may indicate a decrease in the efficacy of the Rph14 gene under the influence of fungicides.

The development of resistance for pathogens often resembles the effect of vertical resistance: in the first years of fungicide use, complete suppression of infection is observed; over time, the emergence of individual tolerant strains and their accumulation in the population is observed; and, finally, there is a complete loss of fungicide efficacy (Dyakov, 1998). One of the factors reducing the sensitivity of phytopathogens to the active substances of preparations is a change in their intrapopulation structure (Tyuterev, 2001). Mutations that cause resistance of phytopathogen isolates to fungicides can lead to a decrease in their adaptability and virulence (Hawkins, Fraaije, 2018), but later, an increase in the aggressiveness of the pathogen may be observed (Dyakov, 1998). In our studies for the North Caucasian population of the barley leaf rust pathogen under the influence of two-component preparations of the class of triazoles and strobilurins, changes in the intrapopulation structure in terms of aggressiveness and virulence were noted, which justifies the need for constant study of this issue to control the accumulation of resistant forms of P. hordei in the fungal population

## Conclusion

A comparative assessment of pathogenicity indicators under the influence of fungicides of the chemical classes of triazoles and strobilurins revealed a decrease in the aggressiveness and virulence of the North Caucasian population of the barley leaf rust pathogen. So, in all options with an increase in the rate of fungicide application, a change in the intrapopulation structure and average virulence of populations was noted. In all options with an increase in the rate of fungicide application, there was a decrease in the virulence of the fungal population to Rph4, Rph5, Rph6+2, Rph12; in the option with Amistar Extra, SC and Delaro, SC, to Rph2; with Amistar Gold, SC and Amistar Extra, SC, to Rph9.

The use of the studied fungicides at a rate of 50–150 % of the recommended one contributed to an increase in the virulence of the population to Rph14. The line with the Rph7 gene showed no signs of infection both in the original population and in the experimental options. The average virulence of the fungal populations treated with the fungicides in all experimental options was lower compared to the original population (no treatment) (48.5 %). Significant changes in this indicator were noted under the influence of the fungicide Amistar Gold, SC, the average virulence decreased from 33.8 to 28.5 %.

In comparison with the original population (no treatment), an increase in the duration of the latent period of the disease was noted under the influence of high application rates of the preparations. In the Delaro, SC and Amistar Gold, SC options at an application rate of 200 % of the recommended one, the value of this indicator varied from 168 to 216 hours, with Amistar Extra, SC, from 168 to 192 hours.

A decrease in the sporulating capacity of the P. hordei population treated with the fungicides was determined. In the options with the Delaro, SC and Amistar Gold, SC preparations with an increase in the application rate, the spore mass from one pustule decreased from 0.013 (original population (no treatment)) to 0.002 mg (application rate 200 %), in the Amistar Extra, SC option, to 0.005 mg (application rate 200 %). A significant decrease in the viability of P. hordei spores in comparison with the original population (no treatment) was noted under the influence of the fungicide Amistar Gold, SC. With an application rate of the preparation of 200 % of the recommended one, this indicator decreased from 100 to 22.5 %

Thus, our studies allow us to identify changes in the population structure by virulence and aggressiveness of the P. hordei population under the influence of the studied fungicides, which will make it possible to promptly adjust the winter barley protection system and, in the future, can contribute to the development of an anti-resistant strategy to control P. hordei.

## Conflict of interest

The authors declare no conflict of interest.
